# Child stunting prevalence determination at sector level in Rwanda using small area estimation

**DOI:** 10.1186/s40795-023-00806-w

**Published:** 2023-12-12

**Authors:** Innocent Ngaruye, Joseph Nzabanita, François Niragire, Theogene Rizinde, Joseph Nkurunziza, Jean Bosco Ndikubwimana, Charles Ruranga, Ignace Kabano, Dieudonne N. Muhoza, Jeanine Ahishakiye

**Affiliations:** 1 Department of Mathematics, College of Science and Technology, University of Rwanda, Kigali, Rwanda; 2 Department of Applied Statistics, College of Business and Economics, University of Rwanda, Kigali, Rwanda; 3 African Centre of Excellence in Data Science, College of Business and Economics, University of Rwanda, Kigali, Rwanda; 4 Department of Human Nutrition and Dietetics, College of Medicine and Health Sciences, University of Rwanda, Kigali, Rwanda

**Keywords:** Generalized linear mixed model, Small area estimation, Stunting, Undernutrition

## Abstract

**Background:**

Stunting among children under 5 years of age remains a worldwide concern, with 148.1 million (22.3%) stunted in 2022. The recent 2019/2020 Rwanda Demographic Health Survey (RDHS) revealed that the prevalence of stunting in Rwanda among under five children was 33.5%. In Rwanda, there is no sufficient evidence on stunting status to guide prioritized interventions at the sector level, the lowest administrative unit for implementing development initiatives. This study aimed to provide reliable estimates of stunting prevalence in Rwanda at the sector level.

**Methods:**

In this article, Small Area Estimation (SAE) techniques were used to provide sector level estimates of stunting prevalence in children under five in Rwanda. By plugging in relevant significant covariates in the generalized linear mixed model, model-based estimates are produced for all sectors with their corresponding Mean Square Error (MSE).

**Results:**

The findings showed that, overall, 40 out of 416 sectors had met the national target of having a stunting rate less than or equal to 19%, while 194 sectors were far from meeting this target, having a stunting rate higher than the national prevalence of 33.5% in the year 2020. The majority of the sectors with stunting prevalence that were higher than the national average of 33.5% were found in the Northern Province with 68 sectors out of 89 and in Western Province with 64 sectors out of 96. In contrast, the prevalence of stunting was lower in the City of Kigali where 14 out of 35 sectors had a stunting rate between 0 and 19%, and all sectors were below the national average. This study showed a substantial connection between stunting and factors such as household size, place of residence, the gender of the household head, and access to improved toilet facilities and clean water.

**Conclusion:**

The results of this study may guide and support informed policy decisions and promote localised and targeted interventions in Rwanda’s most severely affected sectors with a high stunting prevalence in Rwanda.

**Supplementary Information:**

The online version contains supplementary material available at 10.1186/s40795-023-00806-w.

## Introduction

Child undernutrition is among important global development challenges, and reducing it has remained among the key global priorities. Stunting in children under 5 years of age continues to be a worldwide concern especially in developing countries. According to the World Health Organization, there were 148.1 million (22.3%) stunted children under five of age in 2022 [1]. Children are stunted if their length/height is below -2 standard deviations (SD) from the World Health Organization (WHO) child growth Standards median for the same age and sex [2]. Africa was the only region where the number of stunting among under five increased over the past decade, from 54.4 million in 2000 to 61.4 million in 2020 [3].

Stunted children can suffer severe, irreversible physical and cognitive damage that accompany stunted growth [2, 4]. These children are at a disadvantage of facing learning difficulties in school, decreased productivity at work and facing barriers to maximize their participation in their communities [2, 4]. Thus, this affects the society as a whole and can even affect the next generation [2, 4, 5]. Addresing these stunting realated concerns are consistent with the target of the global Sustainable Development Goal (SDG) 2 aiming to significantly reduce child stunting by 2025 [6].

In Rwanda, the national average stunting rate in children under 5 years of age declined from 44% in 2010 to 33% in 2020 [3, 7]. However, this rate is still higher than the national and global targets and remains higher than the 2020 average for the Africa region (30.7%) [2]. Disparities in child stunting have also remained prevalent in Rwanda. District level stunting prevalence have been unevenly distributed and within the same district, the stunting prevalence may also vary substantially. For example 10.7% of children under five in Kicukiro district are stunted , while 50.5% of children in Ngororero district in 2020 [4].

The Government of Rwanda through the National Institute of Statistics of Rwanda conducts the Rwanda Demographic and Health Surveys (RDHS) every 5 years. The RDHS designed to collect demographic, health, and nutrition information in developing countries, it provides estimates of stunting prevalence at national, province and district levels lacking administrative sector level estimates of under-nutrition indicators, such as stunting [2, 3, 8]. As a consequence, the direct estimates of childhood stunting are with high variability and thus not reliable due to small samples connected to a sector.

This translates into insufficient evidence needed to appropriately guide policy making processes as decentralized decision making necessitate decision makers having access to estimates for small geographic areas. Also, it results in waste of resources due to lack of evidence to guide highly prioritized interventions. Therefore, there is a need for more improved understanding of the dynamics of child undernutrition as well as a more disaggregated child nutrition information, especially at the levels of implementation to enable prioritized and targeted interventions [4, 9–12]. Therefore, the current paper aims at providing sector level estimates of stunting prevalence in children under five in Rwanda using Small Area Estimation (SAE) techniques, to guide policy making and support localized and targeted interventions to the most affected sectors. The basic idea of SAE techniques is to borrow strength from other relevant domains in the same area and improve the precision and accuracy of direct estimates at a sub-population level.

To the best of our knowledge, this study is the first to provide accurate estimates of stunting prevalence in Rwanda at sector level using RDHS data.

## Methods

### Study setting

This study was conducted in Rwanda. Formally the Republic of Rwanda, is a landlocked country in Africa’s Great Rift Valley, where the African Great Lakes region and East Africa meet. Rwanda is bordered by Uganda, Tanzania, Burundi, and the Democratic Republic of the Congo, and is located a few degrees south of the Equator. Its landscape is dominated by mountains in the west and savanna in the east, with numerous lakes throughout the country, earning it the nickname “land of a thousand hills”. The climate ranges from temperate to sub-tropical, with two rainy and two dry seasons per year. Rwanda is the most densely populated mainland African country, with a population of over 12.6 million people living on $26,338 km^2$ of territory. Kigali, is the capital and largest city of Rwanda, with a population of more than one million people.The Rwandan population is primarily rural and young.

#### Research design and data description

This study used the Rwanda Demographic and Health Surveys of 2019/2020 (RDHS) which is a large and rich dataset on stunting and socio-demographic characteristics. The 2019/2020 RDHS used a two stage sample design for a number of constrained indicators, allowing estimates of significant indicators for the country as a whole, as well as for urban and rural areas, five provinces, and each of Rwanda’s 30 districts. The initial step was to select randomly a sample of 500 clusters made up of EAs defined for the Fourth Rwanda Population and Housing Census (RPHC-2012). The second stage involved households sampling in a every sampled cluter by systematic manner. From June to August 2019, a household listing operation was conducted in all selected EAs, and a total of 13,000 households were selected. RDHS provides the variables as well as the total population and under five years children for only selected sectors while the Small Area Estimates (SAEs) technique are used to produce sector-level estimates of stunted children in all Rwandan sectors for the year 2019/2020. The lack of total children under five years in RDHS for all sector is addressed by a projection based on the RPHC of 2012. The variables of interest considered in this study are:

- The **Response variable** is the Number of stunted children in a sector obtained from RDHS-2019/2020. A stunted child refers to a child who is too short for his or her age. The World Health Organization (WHO) defines a stunted child as a child whose height-for-age Z-score is below minus 2 standard deviations (SD) from the WHO Child Growth Standards median [13]. In this definition, the WHO uses a statistical measure called the Z-score, which indicates how much a child’s height for age deviates in standard deviations from the median of a reference population.
- The sector level **Covariates** were selected from RPHC-2012 which are Proportion of Poverty Headcount, Average Household size, Number of under five children living in urban settlement, Number of male headed households, Number of female headed households, Number of heads of households who completed secondary education level and above, Number of heads of households who have low than secondary education level, Number of households who have access to improved water and Number of households who have access to improved toilet

### Study population

The Rwanda 2019/2020 DHS is one of the accurate and legitimate source of data that was available and accessible, and it could better answer to the study’s goal. All children born in the five years leading up to the 2019/2020 RDHS are included in the study population. The current study’s sample includes all children aged 0 to 59 months at the time of the survey interviews, as well as their mothers, and the necessary anthropometric measurements (sex, height, and age) were obtained during the surveys studied. The total number of children under the age of five that are eligible for this study in the 2019/2020 RDHS is 8,092. The methodology employed in the RDHSs in the report is thoroughly documented [3].

### Method of data analysis

Since the RDHS-2019/2020 was not designed to provide estimates of under five children at a lower level than the district, the direct estimates of childhood stunting are with high variability and thus not reliable due to small samples connected to a sector. Therefore, SAE techniques were used to produce accurate estimates of childhood stunting prevalence at sector level. This method consists of linking statistical models to the variables of interest together with relevant covariates to produce model-based estimates at domain level of interest. A generalized linear mixed model will be considered.

Let $N_i$ and $n_i$ be the population and sample sizes in the sector $i (i=1,...,d)$, respectively, where $d=416$ sectors in the population. The population $N_i$ stands for the total number of children under 5 years in the i-th sector, while the sample size $n_i$ represents the number of children sampled from the i-th sector to participate in RDHS-2019/2020. The total number of units in the population (children under 5) is $N=N_1+N_2+...+N_d$ and the total sample *n* is $n=n_1+n_2+...+n_k$. Moreover, The response variable $y_{ij}$ that takes the value of the j-th child, in the i-th sector is a binary random variable that takes the value 1 if a child is stunted and 0 if a child is not. In addition, the response vector $\textbf{y}_{i}$ for the i-th sector is partitioned into sampled $\textbf{y}_{i}^s=\left( y_{ij}^s\right),~j=1,...,n_i$ and non-sampled $\textbf{y}_{i}^r=\left( y_{ij}^r\right),~j=n_{i}+1,...,N_i$ parts. It follows that the total number of stunted children in sector *i* is $T_i=T_i^s+T_i^r$, with $T_i^s=\sum _{j=1}^{n_i}y_{ij}^s$ and $T_i^r=\sum _{i}^{N_i-n_i}y_{ij}^r$, where $T_i^s$ and $T_i^r$ are independent variables assumed to follow a Poisson distribution. Let $\varvec{x}_{ij}$ be a vector of covariates. The linking model to covariates is a log linear model of the form

1 $$\begin{aligned} \log (T_i)=\varvec{x}_{ij}'\varvec{\beta }+u_i, \end{aligned}$$

where, $\varvec{\beta }$ is a k-vector of unknown parameters, and $u_i\sim \mathcal {N}(0, \sigma ^2)$ is the random effect that accounts for the between variability other than the variability explained by the covariates included in the model. The estimators of unknown parameters $\varvec{\beta }$ and $u_i$ from model (1), can be obtained using maximum likelihood approach using the sample data and the total number of stunted children in the i-th sector [14]:

2 $$\begin{aligned} \nonumber \widehat{T}_i={} & {} T_i^s+\widehat{T}_i^r\\ \widehat{T}_i={} & {} T_i^s+(N_i-n_i)\ \textrm{exp}\left( \varvec{x}_{ij}'\widehat{\varvec{\beta }}+\widehat{u}_i\right) , \end{aligned}$$

where $T_i^s$ is straightforward calculated from the sample data. The proportion $p_i$ of stunted children in the i-th sector is estimated as

3 $$\begin{aligned} \widehat{p}_i= \frac{\widehat{T}_i}{N_i}. \end{aligned}$$

Here, $N_i$ is computed using the following formula

$$\begin{aligned} N_i=pN_{i,Tot}, \end{aligned}$$

where *p* and $N_{i,Tot}$, the proportion of under five children in the population and the total population of the i-th sector in 2019, are computed using medium population projection [15].

It should be noted that model (1) is based on an unweighted sample count, which assumes that sampling within areas is non-informative [16]. Therefore, Eq. (2) disregards the complex survey design such as Demographic Health Survey. In order to address this issue, several authors suggested that when analyzing area level estimates as a binomial proportion, one should use the *effective sample size* by incorporating sampling weights rather than the actual sample size in the model [17, 18]. Sampling weights are necessary in complex sampling designs like the stratified two-stage cluster design used for demographic health surveys in order to account for the complex sampling design, variations in selection probabilities, and potential biases introduced by non-response or other factors.

Let $N_{ih}$ and $n_{ih}$ denote the population size and sample size in cluster *h* in area *i*, respectively, such that $N_i=\sum _hN_{ih}$ and $n_i=\sum _hn_{ih}$ and the associated sampling weights noted by $w_{ih}=\frac{N_{ih}}{n_{ih}}$. Define $y_{ijh}$ to be the binary response for the characteristic of interest for unit *j* in cluster *h* in area *i*. The sample size $n_i$ is replaced by effective sample size $n_{i(e)}$ in Eq. (2) as proposed by Liu et al. [18], where

$$\begin{aligned} n_{i(e)}=\frac{P_i(1-P_i)}{n_i\text {Var}(p_{iw})}Deff_i, \end{aligned}$$

for small area proportion $P_i$

$$\begin{aligned} P_i=\frac{\sum _h\sum _jy_{ijh}}{N_{jh}}, \end{aligned}$$

whose direct survey estimator is given by

$$\begin{aligned} p_{iw}=\frac{\sum _h\sum _jw_{ih}y_{ijh}}{\sum _h\sum _jw_{ih}}, \end{aligned}$$

where

$$\begin{aligned} Deff_i=\frac{W_{ih}^2P_{ih}(1-P_{ih})/n_{ih}}{P_i(1-P_i)/n_i} \end{aligned}$$

for $W_{ih}=\frac{N_{ih}}{N_i}$ and population proportion in cluster *h* in area *i* given by $P_{ih}$. Moreover, the design count $\widehat{T}_i^s$ is replaced by effective count $\widehat{T}_{i(e)}^s$ in (2) defined by

$$\begin{aligned} \widehat{T}_{i(e)}^s=n_{i(e)}p_{iw}. \end{aligned}$$

Therefore, total number of stunted children in the i-th sector is computed as

4 $$\begin{aligned} \widehat{T}_i=T_{ie}^s+(N_i-n_{ie})\ \textrm{exp}\left(\varvec{x}_{ij}'\widehat{\varvec{\beta }}+\widehat{u}_i\right) \end{aligned}$$

Usually, for Demographic Health Survey (DHS), a stratified two-stage cluster design is used to make the sample. Enumeration Areas (EA) are typically selected from census files in the first stage. In the second stage, a sample of homes is drawn from an updated list of households in each EA that was selected. Given *H* strata, let $y_{hi}$ be the response value for a characteristic of interest for *i*-th sampling unit in the *h*-th stratum, $w_{hi}$ be the corresponding sampling weight, and $n_{h}$ the sample size of the *h*-th stratum. The estimator of the population mean is the weighted mean given by

$$\begin{aligned} \bar{y}=\frac{\sum _{h=1}^H\sum _{i=1}^{n_h}w_{hi}y_{hi}}{\sum _{h=1}^H\sum _{i=1}^{n_h}w_{hi}}. \end{aligned}$$

The corresponding sampling variance estimator and standard error (SE) of the weighted mean can be expressed as

$$\begin{aligned} \widehat{\textrm{var}}(\bar{y})={} & {} \frac{\sum _{h=1}^H\frac{n_h}{n_h-1}\sum _{i=1}^{n_h}\Big [w_{hi}(y_{hi}-\bar{y})-\frac{1}{n_h}\sum _{j=1}^{n_h}w_{hj}(y_{hj}-\bar{y})\Big ]^2}{\Big ( \sum _{h=1}^H\sum _{i=1}^{n_h}w_{hi}\Big )^2}\\ SE(\bar{y})={} & {} \sqrt{\widehat{\textrm{var}}(\bar{y})} \end{aligned}$$

## Results

On the first stage, all possible covariates associated with stunting were selected based on literature review. Then, using the Restricted Maximum likelihood (REML) method of estimation, the significant covariates to be included in the final model were selected by testing the significance of the estimates of the fixed effect parameters of the fitted model. In this study, the analysis was carried out using R software. The following Table 1 displays the estimated fixed effect parameters together with the standard errors and corresponding p-values for the final selected model. This table also shows seven covariables that were significantly correlated to the response variable, namely the size of the household, type of residence (urban or rural), sex of household head, household with improved water or not and household with improved toilet or not.

**Table 1:** Estimate of fixed effect parameters of the fitted model

Coefficients	Estimate	Std.Error	z-value	$Pr(>|z|)$
(Intercept)	3.292e+01	1.588e-01	20.731	$$< 2e-16^{***}$$
Prop. Poverty Headcount	-8.148e-05	7.844e-04	-0.104	0.917275
Prop. HH size	6.326e-02	2.786e-02	2.271	$0.023162^{*}$
Count urban	-4.396e-04	1.452e-04	-3.027	$$0.002466^{**}$$
Count rural	-5.072e-04	1.415e-04	-3.583	$$0.000340^{***}$$
Count Male HH head	7.887e-04	3.349e-04	2.355	$0.018534^{*}$
Count HH with improved water	-1.363e-04	2.243e-05	-6.076	$$1.23e-09^{***}$$
Count HH with improved toilet	5.500e-04	1.415e-04	3.887	$$0.000101^{***}$$

It was discovered that during the RDHS, 44 sectors had no sampled clusters, making the sample size zero. One of the 372 sectors with at least one sampled cluster had a direct estimate of the prevalence of stunting equal to 100%, and 43 of them had a direct estimate of the prevalence of stunting equal to zero, demonstrating how far off from the true proportions the direct estimates are. From the RDHS data, direct estimates were mapped to each one 416 sectors in Rwanda. Using generalized linear mixed model as a linking model to auxiliary variables, model-based estimates were computed and both direct and model-based estimates of stunting prevalence of all sectors are presented in Table A.1 in Supplementary files together with the starndard errors and MSE, respectively. It worths to note that the model-based estimates, which were produced by improving the unreliable direct estimates by incorporating relevant auxiliary data and accounting for sector random effect and time variations, are consistent with the direct estimates from RDHS in terms of ranking sector per prevalence of stunting.

**Fig. 1:**
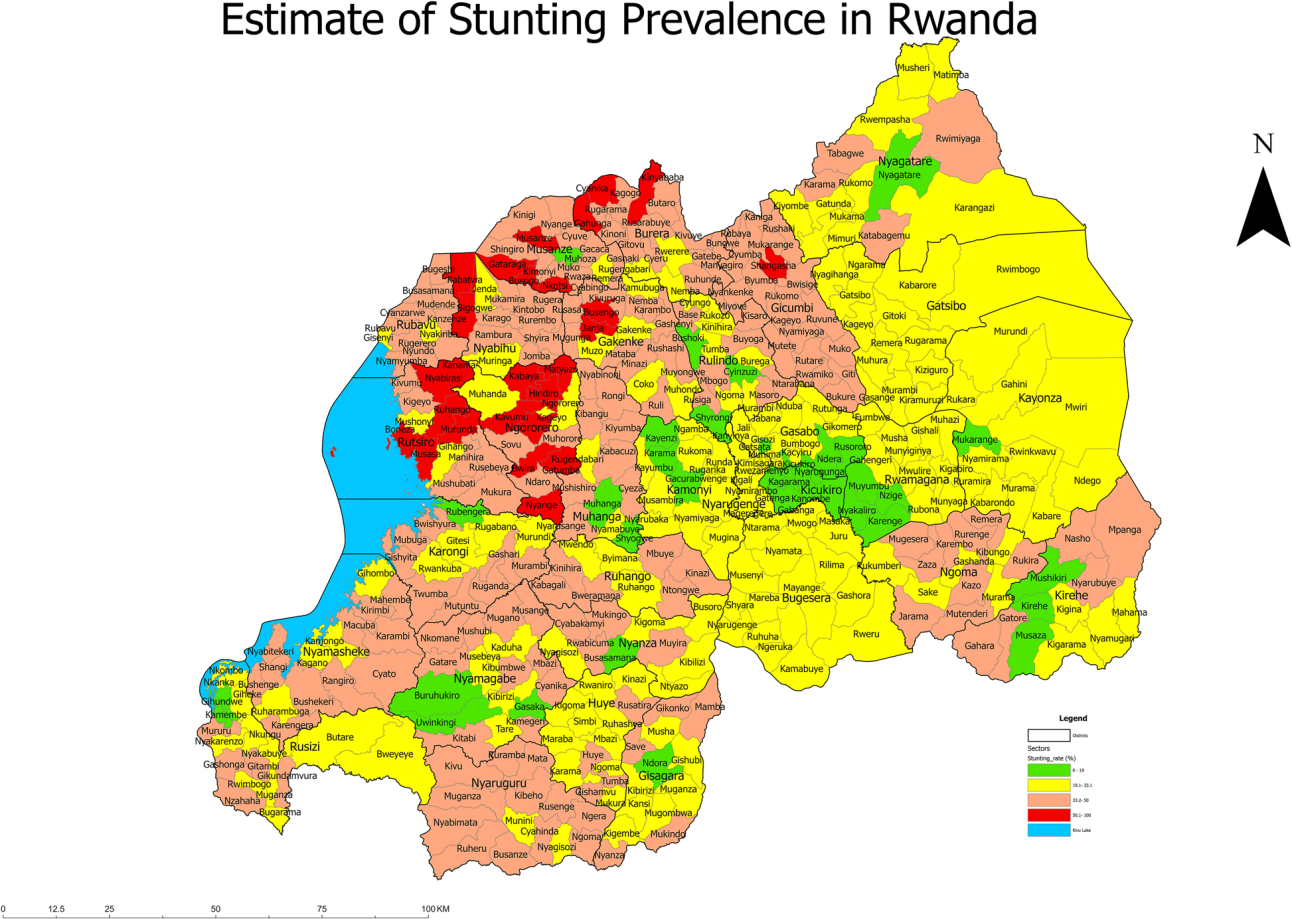
Estimate of Stunting Prevalence in Rwanda

The map in Fig. 1 shows the distribution of national stunting prevalence per sector during year 2019-2020 using model-based estimates. The map in Figs. A.1, A.2, A.3, A.4, and A.5 in Supplementary files, show the distribution of provincial stunting prevalence per sector during year 2019-2020 using model-based estimates.

At national level, the weighted prevalence of stunting was calculated, as direct estimate and it is 33.5%, with SE of 0.8% with 95% confidence interval of [32% –35%]. Table A.1 in Appendix shows that not all sectors have the direct estimates of stunting prevalence, as 44 sectors out of 416 were not sampled. The model-based estimates for the prevalence of stunting in Table A.1 indicated that 40 sectors out of 416 have achieved the national target of having a stunting rate less or equal to 19%, while 194 sectors are far away of achieving this target, having a stunting rate greater than the national prevalence of 33.5%.

Figure 1 shows that most of the sectors with stunting prevalence that were higher than the national average of 33.5% were located in the Western Province with 64 sectors out of 96 and the Northern Province with 68 sectors out of 89. On the other side, the City of Kigali has less stunting prevalence because all sectors are below the national average and 14 sectors out of 35 were between 0-19%. Out of Kigali, Eastern is the less stunted province because none of its sectors is above 50% and only 16 sectors out of 95 were between 33.2% and 50%. The Southern province is in the average situation because none of its sectors is in red color but few of the sectors are in green and most of the sectors are between 33.2% and 50%.

## Discussion

From 2010 to 2020, Rwanda was able to reduce the rate of stunting by around 11% and 5% in ten and five years, respectively. However, compared to the level of Africa and the national target of a 4% annual reduction in stunting, the prevalence of stunting is still high.

Despite all the efforts made by the Government of Rwanda to eradicate undernution among children underfive years of age, the stunting prevalence in Rwanda is still high with 33.5% in 2020 [3]. This study aimed to translate the estimates of stunting prevalence in Rwanda from national and district levels to sector level by using Small Area Estimation techniques applied to 2019-2000 RHDS data. The proportion of the size of household, the place of residence, the sex of the head of household, the source of water and the type of toilet facilities were found to be the most significant risk factors associated to stunting of under 5 children in Rwanda.

The present study’s results revealed that toilet facilities were among key factors associated with stunting in Rwanda, which are in agreement with other previous studies [19, 20]. Furthemore, it is also consistent with the research conducted by Bigirimana [21] that revealed a significant correlation between stunting and family planning, as well as household size in Rwanda. The findings of this study are also in line with earlier research that showed a higher proportion of stunted children resided in rural areas than in urban areas [22–24].

A similar study revealed that being male, being of low birth weight, being in the lowest wealth quintile, having a mother with a low level of education, and having a mother who smokes were all related to higher rates of stunting [25]. Furthermore, breastfeeding, antenatal care visits, sharing a toilet, a child’s gender, the household wealth index, and the province a child lives in were also found to be risk factors for stunting in both the eastern and western provinces [19].

The findings on stunting prevalence distribution per sector showed that the majority of the red-colored sectors (those with a high stunting rate) are located in the northern and western provinces, despite the fact that these areas are well known for their high agricultural production due to their volcanic landscape and perpetual annual rainfall. These issues can be explained by high population density, lack of knowledge on how to prepare balanced diet, and the fact that there is increased workload of parents, particularly for mothers in the Northern and Western provinces.

The political will and established policies to improve nutrition at the national level can be transferred to subnational levels such as sector level for implementation [26]. The results of the study showed that, within districts with high stunting prevalence, we could identify the most vulnerable sectors. Therefore, rather than focusing on a district as a whole, these findings can guide policy decisions on supporting localized and targeted nutrition intervention programs to the most affected sectors.

## Conclusion

One of the major barriers to global development remains to be reducing child undernutrition, which continues to be one of the top global concerns. The child growth patterns have the potential to undermine economic prospects and the nation’s rapid growth trajectory since they are reliable indicators of the health and productivity of future generations as well as the development of human capital. In terms of enhancing child survival, decreasing poverty, raising agricultural output, and enhancing environmental health, Rwanda has obtained significant achievements. However, the nutrition status of young children continues to be a significant outlier, necessitating increased Rwandan efforts.

All of the research findings that are available have either generated national, provincial, or district estimates of stunting but not at sector level. But, in Rwanda, the sector is the third-level administrative subdivision responsible for the implementation of development initiatives, providing services, and promoting social welfare and good governance. The policymakers cannot be informed or guided by these estimates when planning and allocating funds at the sector level. In this article, Small Area Estimation approaches were employed to produce sector-level estimates of the prevalence of stunting in children under five in Rwanda, which can inform and guide policy decisions as well as enable localized and focused nutrition interventions and planning programs at the sector level. Strengthening intervention programs that instruct in people how to prepare a healthy, balanced diet is necessary, particularly in north and south western regions of the country.

### Supplementary Information

**Additional file 1.** This manusrcipt has separate accompying supplementary files.

## Data Availability

All data used in this study come from the Rwanda Demographic Health Surveys found on the link. https://dhsprogram.com/data/available-datasets.cfm
