# Supplementary material for: Child stunting prevalence determination at sector level in Rwanda using small area estimation
Source: BMC Nutr. 2023 Dec 12;9:147. doi: 10.1186/s40795-023-00806-w (PMC10714628; doi:10.1186/s40795-023-00806-w)
Supplement: Supplementary file 1 — Additional file 1. This manusrcipt has separate accompying supplementary files. [file 40795_2023_806_MOESM1_ESM.zip › Supplement_Table_A1.pdf]

Tab.A 1 Stunting prevelance at sector level

Sector code	District	Sector	Direct estimates (%)	SE	Model based estimates (%)	MSE
1101	Nyarugenge	Gitega	30.4	15.6	27.4	6.8
1102	Nyarugenge	Kanyinya	40	21.9	29.5	4.4
1103	Nyarugenge	Kigali	20.1	7.9	24.2	4.1
1104	Nyarugenge	Kimisagara	32.7	9	27.9	6.6
1105	Nyarugenge	Mageragere	35.8	11.9	28.6	3.4
1106	Nyarugenge	Muhima	48.3	10.3	31	7.3
1107	Nyarugenge	Nyakabanda	11.3	10.6	29.7	6.3
1108	Nyarugenge	Nyamirambo	44.5	16.2	30.3	4.4
1109	Nyarugenge	Nyarugenge	39.9	10.1	19.8	8.6
1110	Nyarugenge	Rwezamenyo	33.3	19.2	28.1	10.1
1201	Gasabo	Bumbogo	60	15.5	29.8	4.6
1202	Gasabo	Gatsata	50	20.4	28.4	5
1203	Gasabo	Gikomero	33.3	15.7	25.5	5.4
1204	Gasabo	Gisozi	29.4	14.5	13.6	4.6
1205	Gasabo	Jabana	20	17.9	21.9	3.4
1206	Gasabo	Jali	48.1	11.5	28.1	3.7
1207	Gasabo	Kacyiru	40	21.9	26.9	5.7
1208	Gasabo	Kimihurura	22.2	13.9	22.8	5.7
1209	Gasabo	Kimiromko	31.8	14.8	25.2	7.1
1210	Gasabo	Kinyinya	42.4	12.8	27.2	3.4
1211	Gasabo	Ndera	8.3	8	16.2	4.5
1212	Gasabo	Nduba	53.8	13.8	28.9	3.8
1213	Gasabo	Remera	7.6	7.4	15.7	5.8
1214	Gasabo	Rusororo	0	-	13.6	3.7
1215	Gasabo	Rutunga	0	-	24.6	3.5
1301	Kicukiro	Gahanga	33.1	12.1	10.1	3.5
1302	Kicukiro	Gatenga	22.6	10	9	5.3
1303	Kicukiro	Gikondo	41.7	14.2	10.7	4.7
1304	Kicukiro	Kagarama	66.7	12.2	12.1	6.7
1305	Kicukiro	Kanombe	28.8	9.1	9.7	6
1306	Kicukiro	Kicukiro	28.6	17.1	9.7	5.6
1307	Kicukiro	Kigarama	26.3	7.6	9.4	7.3
1308	Kicukiro	Masaka	52.9	11.5	11.4	4.6
1309	Kicukiro	Niboye	33.3	19.2	10.1	10.6
1310	Kicukiro	Nyarugunga	59.9	14.3	11.8	9.6
2101	Nyanza	Busasamana	27.5	10	17.8	5.2
2102	Nyanza	Busoro	23.9	10.3	30.6	3.2
2103	Nyanza	Cyabakamyi	49.1	13	37.3	2.7
2104	Nyanza	Kibirizi	20	17.9	28.9	1.9
2105	Nyanza	Kigoma	23.1	11.7	30.2	2.6
2106	Nyanza	Mukingo	56	11.1	38.6	5.6
2107	Nyanza	Muyira	55.6	14.5	38.4	2.1
2108	Nyanza	Ntyazo	0	-	31.9	2.3
2109	Nyanza	Nyagisozi	23.7	10.4	30.5	3.7
2110	Nyanza	Rwabicuma	37.5	17.1	34.7	2.6
2201	Gisagara	Gikonko	50	17.7	38.5	3.5
2202	Gisagara	Gishubi	22.5	12.7	30.8	2.1
2203	Gisagara	Kansi	Not sampled	-	32.6	2.5
2204	Gisagara	Kibirizi	25	15.4	31.8	2.2
2205	Gisagara	Kigembe	Not sampled	-	32.6	2.1
2206	Gisagara	Mamba	33.5	11.2	34.6	1.9
2207	Gisagara	Muganza	25	15.3	31.7	2.3
2208	Gisagara	Mugombwa	0	-	27.9	1.9
2209	Gisagara	Mukindo	33.3	19.2	34.5	2.5
2210	Gisagara	Musha	14.3	13.2	26.5	1.7

Sector code	District	Sector	Direct estimates (%)	SE	Model based estimates (%)	MSE
2211	Gisagara	Ndora	16.7	10.8	18.3	2
2212	Gisagara	Nyanza	50	12.5	38.5	1.5
2213	Gisagara	Save	29.5	14	33.3	1.6
2301	Nyaruguru	Busanze	57.8	14.3	45	1.9
2302	Nyaruguru	Cyahinda	44.4	16.6	42.1	1.6
2303	Nyaruguru	Kibeho	27.5	13.5	36.9	1.4
2304	Nyaruguru	Kivu	20	17.9	33.4	2.8
2305	Nyaruguru	Mata	33.3	19.2	38.9	2.1
2306	Nyaruguru	Muganza	37.5	17.1	40.3	1.8
2307	Nyaruguru	Munini	0	-	20.7	1.4
2308	Nyaruguru	Ngera	57.1	18.7	44.9	1.6
2309	Nyaruguru	Ngoma	40	21.9	41	1.5
2310	Nyaruguru	Nyabimata	71.4	17.1	47.3	2.8
2311	Nyaruguru	Nyagisozi	0	-	20.1	1.7
2312	Nyaruguru	Ruheru	30.8	12.8	38	3.1
2313	Nyaruguru	Ruramba	60	21.9	45.5	2.2
2314	Nyaruguru	Rusenge	33.3	15.7	38.9	1.7
2401	Huye	Gishamvu	66.7	27.2	35.6	1.7
2402	Huye	Huye	50	17.7	33.1	2.1
2403	Huye	Karama	Not sampled	-	30.9	3
2404	Huye	Kigoma	33	10.2	29.6	2
2405	Huye	Kinazi	33.3	27.2	29.7	2.6
2406	Huye	Maraba	46.2	13.8	32.5	2
2407	Huye	Mbazi	23.2	15	26.7	2.8
2408	Huye	Mukura	40	15.5	31.2	3.1
2409	Huye	Ngoma	25	12.5	22.9	4.9
2410	Huye	Ruhashya	14.3	13.2	27.4	1.8
2411	Huye	Rusatira	75	21.7	36.6	4.6
2412	Huye	Rwaniro	16.7	15.2	24.1	2.2
2413	Huye	Simbi	25	15.3	27.4	2.5
2414	Huye	Tumba	51	15.8	33.3	4.5
2501	Nyamagabe	Buruhukiro	0	-	18.7	1.5
2502	Nyamagabe	Cyanika	40	15.5	38	2.2
2503	Nyamagabe	Gasaka	0	-	18.7	2
2504	Nyamagabe	Gatare	85.7	13.2	45.8	1.5
2505	Nyamagabe	Kaduha	20	12.7	31	1.6
2506	Nyamagabe	Kamegeri	40	21.9	38	2
2507	Nyamagabe	Kibirizi	21.4	11	31.7	1.8
2508	Nyamagabe	Kibumbwe	Not sampled	-	36	2.2
2509	Nyamagabe	Kitabi	40	21.9	38	2.2
2510	Nyamagabe	Mbazi	50	17.7	40.4	4.6
2511	Nyamagabe	Mugano	50	15.8	40.3	2.9
2512	Nyamagabe	Musange	71.4	17.1	44	2.3
2513	Nyamagabe	Musebeya	20	12.7	31	2.4
2514	Nyamagabe	Mushubi	Not sampled	-	35.9	2.2
2515	Nyamagabe	Nkomane	28.6	17.1	34.6	2.3
2516	Nyamagabe	Tare	22.3	11.5	32.2	1.5
2517	Nyamagabe	Uwinkingi	0	-	18.7	2.2
2601	Ruhango	Bweramana	63.6	14.5	49.2	2
2602	Ruhango	Byimana	8.7	8.2	26.9	2.9
2603	Ruhango	Kabagali	28	10.8	39.5	2.5
2604	Ruhango	Kinazi	39.7	13.5	43.6	4.2
2605	Ruhango	Kinihira	0	-	40.8	2.5
2606	Ruhango	Mbuye	51.4	12	46.5	2.6
2607	Ruhango	Mwendo	10	9.5	28.1	10.5
2608	Ruhango	Ntongwe	18.2	16.7	34.5	2.4
2609	Ruhango	Ruhango	31.7	8.6	20.5	1.9

Sector code	District	Sector	Direct estimates (%)	SE	Model based estimates (%)	MSE
2701	Muhanga	Cyeza	0	-	33.8	2.2
2702	Muhanga	Kabacuzi	33.3	15.7	34	2.3
2703	Muhanga	Kibangu	37.5	17.1	35.1	3
2704	Muhanga	Kiyumba	36.4	14.5	34.8	2.8
2705	Muhanga	Muhanga	33.2	13.6	17.5	4.6
2706	Muhanga	Mushishiro	36.4	14.5	34.8	4.7
2707	Muhanga	Nyabinoni	Not sampled	-	33.1	3.3
2708	Muhanga	Nyamabuye	24.3	6.5	31.1	3.9
2709	Muhanga	Nyarusange	56.8	15.8	39.1	2.7
2710	Muhanga	Rongi	58.3	14.2	39.3	3.4
2711	Muhanga	Rugendabari	20	17.9	29.2	2.3
2712	Muhanga	Shyogwe	0	-	17.8	2.7
2801	Kamonyi	Gacurabwenge	11.1	10.5	18.1	2.2
2802	Kamonyi	Karama	0	-	13.5	1.8
2803	Kamonyi	Kayenzi	33.3	27.2	18.1	2.4
2804	Kamonyi	Kayumbu	20	12.7	22	2.2
2805	Kamonyi	Mugina	16.9	8.9	29.5	2.2
2806	Kamonyi	Musambira	57.1	13.2	20.7	2.5
2807	Kamonyi	Ngamba	28.6	17.1	24.5	2
2808	Kamonyi	Nyamiyaga	30	12.6	24.9	2.2
2809	Kamonyi	Nyarubaka	14.3	13.2	19.6	2.3
2810	Kamonyi	Rugarika	11.1	10.5	25.6	2.1
2811	Kamonyi	Rukoma	46.9	12.7	28.1	3.1
2812	Kamonyi	Runda	22.7	10.2	22.9	2.6
3101	Karongi	Bwishyura	45	10.4	38.5	3.5
3102	Karongi	Gashari	0	-	34.5	1.5
3103	Karongi	Gishyita	40	21.9	33.3	3.8
3104	Karongi	Gitesi	0	-	29.7	4.5
3105	Karongi	Mubuga	66.7	27.2	38.1	1.6
3106	Karongi	Murambi	33.3	27.2	36.3	1.6
3107	Karongi	Murundi	50	35.4	30.8	1.8
3108	Karongi	Mutuntu	8.3	8	34.1	3.5
3109	Karongi	Rubengera	37	12.6	18.4	1.9
3110	Karongi	Rugabano	14.3	13.2	22	1.9
3111	Karongi	Ruganda	0	-	39.2	1.7
3112	Karongi	Rwankuba	20	17.9	21.3	1.6
3113	Karongi	Twumba	20	12.7	36.9	1.7
3201	Rutsiro	Boneza	66.7	19.2	58.9	1.8
3202	Rutsiro	Gihango	10.5	7.4	26.1	2.6
3203	Rutsiro	Kigeyo	33.3	19.2	49.3	1.8
3204	Rutsiro	Kivumu	18.8	8.8	41.4	2.7
3205	Rutsiro	Manihira	33.3	15.7	49.3	3.5
3206	Rutsiro	Mukura	0	-	34	1.5
3207	Rutsiro	Murunda	40	15.5	51.8	1.8
3208	Rutsiro	Musasa	44.4	17.7	54.9	1.6
3209	Rutsiro	Mushonyi	0	-	26.5	2.5
3210	Rutsiro	Mushubati	12.5	11.7	36.3	1.9
3211	Rutsiro	Nyabirasi	42.9	18.7	52.7	1.7
3212	Rutsiro	Ruhango	42.9	18.7	52.7	2.6
3213	Rutsiro	Rusebeya	12.5	11.7	36.3	1.8
3301	Rubavu	Bugeshi	60	21.9	44.8	3.4
3302	Rubavu	Busasamana	33.3	15.7	38.3	2.1
3303	Rubavu	Cyanzarwe	13.8	10.3	43.7	5.9
3304	Rubavu	Gisenyi	54.2	10.9	29.4	3.9
3305	Rubavu	Kanama	Not sampled	-	40.8	2.9

Sector code	District	Sector	Direct estimates (%)	SE	Model based estimates (%)	MSE
3306	Rubavu	Kanzenze	50	35.4	42.8	2.6
3307	Rubavu	Mudende	66.7	19.2	45.9	1.7
3308	Rubavu	Nyakiriba	20	12.7	33	1.9
3309	Rubavu	Nyamyumba	64.6	10.1	45.6	1.8
3310	Rubavu	Nyundo	Not sampled	-	40.8	2.9
3311	Rubavu	Rubavu	22.5	14	34.3	4.1
3312	Rubavu	Rugerero	33.3	27.2	38.4	2
3401	Nyabihu	Bigogwe	43	18.7	52.4	2.4
3402	Nyabihu	Jenda	0	-	26.3	1.8
3403	Nyabihu	Jomba	25	21.7	44.9	2.1
3404	Nyabihu	Kabatwa	41.7	14.2	52	5.7
3405	Nyabihu	Karago	18.2	11.6	40.7	2.1
3406	Nyabihu	Kintobo	21.9	11.6	43.2	2.1
3407	Nyabihu	Mukamira	33.4	15.7	48.8	2.1
3408	Nyabihu	Muringa	33.3	15.7	25.3	2
3409	Nyabihu	Rambura	35.6	12.8	49.7	4.1
3410	Nyabihu	Rugera	21.4	11	42.8	3.1
3411	Nyabihu	Rurembo	0	-	48.9	1.6
3412	Nyabihu	Shyira	16.7	15.2	39.5	1.6
3501	Ngororero	Bwira	75	15.3	65.6	2.3
3502	Ngororero	Gatumba	50	17.7	59.4	2
3503	Ngororero	Hindiro	40	21.9	56.1	1.4
3504	Ngororero	Kabaya	50	18.1	59.5	2.8
3505	Ngororero	Kageyo	50	20.4	59.5	1.6
3506	Ngororero	Kavumu	75	21.7	65.6	1.8
3507	Ngororero	Matyazo	44.4	16.6	57.6	2
3508	Ngororero	Muhanda	0	-	27.6	1.9
3509	Ngororero	Muhororo	9.1	8.7	35	1.5
3510	Ngororero	Ndaro	0	-	42.4	1.7
3511	Ngororero	Ngororero	15.8	6.2	27.9	2.1
3512	Ngororero	Nyange	30.8	12.8	52.1	2
3513	Ngororero	Sovu	14.3	13.2	40.9	3.3
3601	Rusizi	Bugarama	29.5	11.8	31.6	3
3602	Rusizi	Butare	25	21.7	30.2	2.6
3603	Rusizi	Bweyeye	Not sampled	-	31.6	3.1
3604	Rusizi	Gashonga	41.7	14.2	34.8	2.9
3605	Rusizi	Giheke	0	-	30.6	3.2
3606	Rusizi	Gihundwe	26.4	11.4	17.5	3.6
3607	Rusizi	Gikundamvura	60	21.9	38.2	3.7
3608	Rusizi	Gitambi	42.9	18.7	35.1	2.6
3609	Rusizi	Kamembe	Not sampled	-	17.3	3.1
3610	Rusizi	Muganza	15.4	10	25.8	3.6
3611	Rusizi	Mururu	35.7	12.8	33.4	4.8
3612	Rusizi	Nkanka	25	12.5	30.1	3.3
3613	Rusizi	Nkombo	Not sampled	-	31.5	3.3
3614	Rusizi	Nkungu	0	-	31.5	2.6
3615	Rusizi	Nyakabuye	33.3	13.6	32.7	2.5
3616	Rusizi	Nyakarenzo	33.3	27.2	32.8	2.9
3617	Rusizi	Nzahaha	40	15.5	34.4	2.4
3618	Rusizi	Rwimbogo	Not sampled	-	31.5	3.1
3701	Nyamasheke	Bushekeri	78.6	11	49.5	4.2
3702	Nyamasheke	Bushenge	Not sampled	-	37.3	3.1
3703	Nyamasheke	Cyato	22.2	13.9	35.4	2.4
3704	Nyamasheke	Gihombo	11.1	10.5	28.1	2.4
3705	Nyamasheke	Kagano	28.3	12.2	21.1	1.4

Sector code	District	Sector	Direct estimates (%)	SE	Model based estimates (%)	MSE
3706	Nyamasheke	Kanjongo	14.3	7.6	30.6	2
3707	Nyamasheke	Karambi	50	17.7	44.3	2.2
3708	Nyamasheke	Karengera	20.2	9.2	34.3	2.3
3709	Nyamasheke	Kirimbi	Not sampled	-	37.3	2.5
3710	Nyamasheke	Macuba	0	-	37.9	1.9
3711	Nyamasheke	Mahembe	44.4	16.6	43	1.8
3712	Nyamasheke	Nyabitekeri	26.7	11.4	37.4	3.2
3713	Nyamasheke	Rangiro	62.5	12.1	46.9	2.4
3714	Nyamasheke	Ruharambuga	11.6	6.7	28.6	2.7
3715	Nyamasheke	Shangi	Not sampled	-	37.2	2.3
4101	Rulindo	Base	53.8	13.8	35.3	1.8
4102	Rulindo	Burega	30	14.5	30.2	2.3
4103	Rulindo	Bushoki	0	-	15.8	3.1
4104	Rulindo	Buyoga	75	12.5	38.3	1.8
4105	Rulindo	Cyinzuzi	0	-	16	2.3
4106	Rulindo	Cyungo	Not sampled	-	32.4	2.3
4107	Rulindo	Kinihira	9.9	5.1	21.2	2.4
4108	Rulindo	Kisaro	60	21.9	36.3	1.8
4109	Rulindo	Masoro	80	17.9	38.9	2.1
4110	Rulindo	Mbogo	75	15.3	38.3	3
4111	Rulindo	Murambi	22.2	13.9	27.6	2.2
4112	Rulindo	Ngoma	Not sampled	-	32.4	2.3
4113	Rulindo	Ntarabana	42.9	18.7	33.3	2
4114	Rulindo	Rukozo	Not sampled	-	32.4	2.3
4115	Rulindo	Rusiga	55.6	16.6	35.6	1.6
4116	Rulindo	Shyorongi	35.9	14.6	16.6	2
4117	Rulindo	Tumba	0	-	31.8	1.8
4201	Gakenke	Busengo	0	-	50.1	1.8
4202	Gakenke	Coko	10	9.5	29.9	1.9
4203	Gakenke	Cyabingo	Not sampled	-	42.6	1.6
4204	Gakenke	Gakenke	55.6	16.6	22.6	1.2
4205	Gakenke	Gashenyi	50	15.8	48.8	1.7
4206	Gakenke	Janja	83.3	15.2	55.2	1.8
4207	Gakenke	Kamubuga	0	-	23.4	1.8
4208	Gakenke	Karambo	Not sampled	-	42.6	1.7
4209	Gakenke	Kivuruga	30	14.5	42.5	1.2
4210	Gakenke	Mataba	28.6	17.1	41.9	1.8
4211	Gakenke	Minazi	Not sampled	-	42.7	1.6
4212	Gakenke	Mugunga	33.3	15.7	43.8	2.6
4213	Gakenke	Muhondo	12.5	11.7	32.2	1.9
4214	Gakenke	Muyongwe	50	17.7	48.8	1.8
4215	Gakenke	Muzo	0	-	23.7	2.2
4216	Gakenke	Nemba	28.3	12	41.8	1.8
4217	Gakenke	Ruli	Not sampled	-	42.6	1.8
4218	Gakenke	Rusasa	54.5	15	49.9	1.9
4219	Gakenke	Rushashi	20	17.9	37.6	3.7
4301	Musanze	Busogo	57.1	18.7	54.7	2.3
4302	Musanze	Cyuve	33.3	13.6	47.5	2.4
4303	Musanze	Gacaca	37.5	17.1	49	1.8
4304	Musanze	Gashaki	Not sampled	-	47.7	2.6
4305	Musanze	Gataraga	60	21.9	55.3	1.6
4306	Musanze	Kimonyi	26.4	11.5	44.4	6.1
4307	Musanze	Kinigi	40	21.9	49.8	1.7
4308	Musanze	Muhoza	61.1	11.5	16.3	4
4309	Musanze	Muko	20	12.7	40.8	2.1

Sector code	District	Sector	Direct estimates (%)	SE	Model based estimates (%)	MSE
4310	Musanze	Musanze	45	14.2	51.5	1.9
4311	Musanze	Nkotsi	8.3	8	55.6	2.2
4312	Musanze	Nyange	Not sampled	-	47.6	2.7
4313	Musanze	Remera	0	-	30.1	8.8
4314	Musanze	Rwaza	14.3	13.2	36.6	2.1
4315	Musanze	Shingiro	36.4	14.5	48.6	1.9
4401	Burera	Bungwe	Not sampled	-	44	2.9
4402	Burera	Butaro	15.7	6.3	37.9	2
4403	Burera	Cyanika	44.9	13.8	51.6	3.2
4404	Burera	Cyeru	Not sampled	-	44	2.7
4405	Burera	Gahunga	52.6	12	53.7	2.1
4406	Burera	Gatebe	14.3	13.2	36.6	1.4
4407	Burera	Gitovu	Not sampled	-	43.9	2.4
4408	Burera	Kagogo	25	21.7	43.8	1.8
4409	Burera	Kinoni	25	21.7	43.8	2.1
4410	Burera	Kinyababa	40	21.9	50	3.8
4411	Burera	Kivuye	Not sampled	-	44	2.5
4412	Burera	Nemba	0	-	24.6	1.5
4413	Burera	Rugarama	23.1	11.7	42.8	2.8
4414	Burera	Rugengabari	0	-	25.4	2.7
4415	Burera	Ruhunde	37.5	17.1	49.2	2.2
4416	Burera	Rusarabuye	Not sampled	-	43.9	2.4
4417	Burera	Rwerere	0	-	25.7	2.4
4501	Gicumbi	Bukure	25	27.2	40.3	2.5
4502	Gicumbi	Bwisige	Not sampled	-	42.2	2.1
4503	Gicumbi	Byumba	20.9	9.5	35	1.6
4504	Gicumbi	Cyumba	Not sampled	-	42.2	2.2
4505	Gicumbi	Giti	25	21.7	37	1.8
4506	Gicumbi	Kageyo	37.5	17.1	41.7	3.7
4507	Gicumbi	Kaniga	25	21.7	37	1.5
4508	Gicumbi	Manyagiro	Not sampled	-	42.2	2.2
4509	Gicumbi	Miyove	40	21.9	42.3	1.6
4510	Gicumbi	Mukarange	25	21.7	37.1	2.1
4511	Gicumbi	Muko	Not sampled	-	42.2	2.1
4512	Gicumbi	Mutete	44.4	16.6	43.6	2.2
4513	Gicumbi	Nyamiyaga	20	17.9	34.7	2.3
4514	Gicumbi	Nyankenke	50	25	44.9	1.6
4515	Gicumbi	Rubaya	Not sampled	-	42.2	2.1
4516	Gicumbi	Rukomo	33.3	13.6	40.3	1.9
4517	Gicumbi	Rushaki	Not sampled	-	42.2	2.1
4518	Gicumbi	Rutare	50	20.4	44.9	3.3
4519	Gicumbi	Ruvune	50	20.4	44.9	2.1
4520	Gicumbi	Rwamiko	Not sampled	-	42.2	2.2
4521	Gicumbi	Shangasha	100	-	52.9	1.6
5101	Rwamagana	Fumbwe	58.9	14.3	27	1.8
5102	Rwamagana	Gahengeri	Not sampled	-	23.3	2.9
5103	Rwamagana	Gishali	71.5	13.8	28.3	2.2
5104	Rwamagana	Karenge	10	9.5	15.8	2.7
5105	Rwamagana	Kigabiro	61.4	10.7	27.3	4.2
5106	Rwamagana	Muhazi	50	25	25.9	3.9
5107	Rwamagana	Munyaga	40	21.9	24.4	1.5
5108	Rwamagana	Munyiginya	Not sampled	-	23.3	2.8
5109	Rwamagana	Musha	20	17.9	20	1.4
5110	Rwamagana	Muyumbu	47.3	13.9	12.8	2.2
5111	Rwamagana	Mwulire	0	-	25.5	6.4

Sector code	District	Sector	Direct estimates (%)	SE	Model based estimates (%)	MSE
5112	Rwamagana	Nyakaliro	9.1	8.7	15.3	2.6
5113	Rwamagana	Nzige	0	-	12.4	2.2
5114	Rwamagana	Rubona	33.3	27.2	23.2	2.1
5201	Nyagatare	Gatunda	12.5	11.7	24.1	1.9
5202	Nyagatare	Karama	42.9	18.7	35	3
5203	Nyagatare	Karangazi	23.6	10.5	29.5	2.6
5204	Nyagatare	Katabagemu	40	15.5	34.3	1.6
5205	Nyagatare	Kiyombe	16.7	15.2	26.4	2.4
5206	Nyagatare	Matimba	Not sampled	-	30.6	2.3
5207	Nyagatare	Mimuri	31.5	10.7	32.2	4.1
5208	Nyagatare	Mukama	14.3	13.2	25	2.1
5209	Nyagatare	Musheri	0	-	31.8	4.3
5210	Nyagatare	Nyagatare	30	14.5	18	3.4
5211	Nyagatare	Rukomo	18.5	10.6	28	1.7
5212	Nyagatare	Rwempasha	Not sampled	-	30.6	2.7
5213	Nyagatare	Rwimiyaga	37.4	10.6	33.7	2.1
5214	Nyagatare	Tabagwe	50	15.8	36.4	5.6
5301	Gatsibo	Gasange	27.3	13.4	23.9	2.3
5302	Gatsibo	Gatsibo	66.7	13.6	30.3	2.8
5303	Gatsibo	Gitoki	75	15.3	31.2	2.6
5304	Gatsibo	Kabarore	14.9	10.5	20.3	2
5305	Gatsibo	Kageyo	20	17.9	21.8	2.2
5306	Gatsibo	Kiramuruzi	50	17.7	28.2	1.7
5307	Gatsibo	Kiziguro	28.6	17.1	24.2	2
5308	Gatsibo	Muhura	49.6	14.2	28.2	1.7
5309	Gatsibo	Murambi	37.5	17.1	26.2	1.7
5310	Gatsibo	Ngarama	Not sampled	-	27.1	2.1
5311	Gatsibo	Nyagihanga	33.3	15.7	25.3	1.7
5312	Gatsibo	Remera	75	21.7	31.1	2.3
5313	Gatsibo	Rugarama	40	12.7	26.6	2
5314	Gatsibo	Rwimbogo	37.5	17.1	26.2	1.9
5401	Kayonza	Gahini	62.5	17.1	31.8	4.8
5402	Kayonza	Kabare	31	10.6	26.4	2.2
5403	Kayonza	Kabarondo	38.5	13.5	28	2.8
5404	Kayonza	Mukarange	62.5	17.1	14.5	3.1
5405	Kayonza	Murama	50	20.4	30	3.1
5406	Kayonza	Murundi	43.4	11.1	28.9	2.2
5407	Kayonza	Mwiri	50	13.4	30.1	3.2
5408	Kayonza	Ndego	0	-	31.8	2.6
5409	Kayonza	Nyamirama	27.3	13.4	25.5	2.1
5410	Kayonza	Rukara	30.6	11.3	26.3	2
5411	Kayonza	Ruramira	60	15.5	31.4	2.6
5412	Kayonza	Rwinkwavu	35.7	12.8	27.5	2.6
5501	Kirehe	Gahara	0	-	37.7	1.9
5502	Kirehe	Gatore	42.9	18.7	34.1	1.8
5503	Kirehe	Kigarama	33.3	13.6	31.9	2.5
5504	Kirehe	Kigina	30	9.5	30.8	5.7
5505	Kirehe	Kirehe	64.1	11.4	17.1	2.7
5506	Kirehe	Mahama	30	14.5	30.9	1.5
5507	Kirehe	Mpanga	54.2	10.2	36.2	1.5
5508	Kirehe	Musaza	0	-	17.1	2
5509	Kirehe	Mushikiri	0	-	17.3	2.9
5510	Kirehe	Nasho	52.7	11.5	36	4
5511	Kirehe	Nyamugari	33.3	15.7	31.9	5.6
5512	Kirehe	Nyarubuye	53.8	13.8	36.1	2.3

Sector code	District	Sector	Direct estimates (%)	SE	Model based estimates (%)	MSE
5601	Ngoma	Gashanda	14.3	13.2	29.9	2.6
5602	Ngoma	Jarama	33.3	19.2	38.8	6.7
5603	Ngoma	Karembo	Not sampled	-	38.7	2.7
5604	Ngoma	Kazo	0	-	39.5	2.5
5605	Ngoma	Kibungo	35.7	11	20.2	2.8
5606	Ngoma	Mugesera	40	15.5	40.7	1.8
5607	Ngoma	Murama	16.7	15.2	31.6	4.2
5608	Ngoma	Mutenderi	57.1	18.7	44.6	1.7
5609	Ngoma	Remera	32.8	10.4	38.5	2.8
5610	Ngoma	Rukira	50	17.7	43.1	2
5611	Ngoma	Rukumberi	11.1	10.5	27.3	1.9
5612	Ngoma	Rurenge	50	25	43.2	1.8
5613	Ngoma	Sake	15.4	10	30.6	3.4
5614	Ngoma	Zaza	75	15.3	47.7	1.9
5701	Bugesera	Gashora	Not sampled	-	27.7	3.1
5702	Bugesera	Juru	60	12.7	32.4	3.2
5703	Bugesera	Kamabuye	20	17.9	23.8	2.3
5704	Bugesera	Mareba	25	15.3	25.5	1.8
5705	Bugesera	Mayange	18.2	11.6	23.2	5.3
5706	Bugesera	Musenyi	10.4	9.8	27.7	2
5707	Bugesera	Mwogo	14.3	13.2	21.5	4.5
5708	Bugesera	Ngeruka	50	15.8	30.9	2.3
5709	Bugesera	Ntarama	14.3	13.2	19.2	2.2
5710	Bugesera	Nyamata	33	11.1	27.7	3.4
5711	Bugesera	Nyarugenge	50	17.7	31	3.5
5712	Bugesera	Rilima	42.2	12.3	29.6	2.3
5713	Bugesera	Ruhuha	30	14.5	26.9	1.5
5714	Bugesera	Rweru	29.5	13.6	26.8	2.6
5715	Bugesera	Shyara	Not sampled	-	27.1	2.9
