# Supplementary figures and images for: Child stunting prevalence determination at sector level in Rwanda using small area estimation
Source: BMC Nutr. 2023 Dec 12;9:147. doi: 10.1186/s40795-023-00806-w (PMC10714628; doi:10.1186/s40795-023-00806-w)

**Fig.A 1** Estimate of Stunting Prevalence in City of Kigali

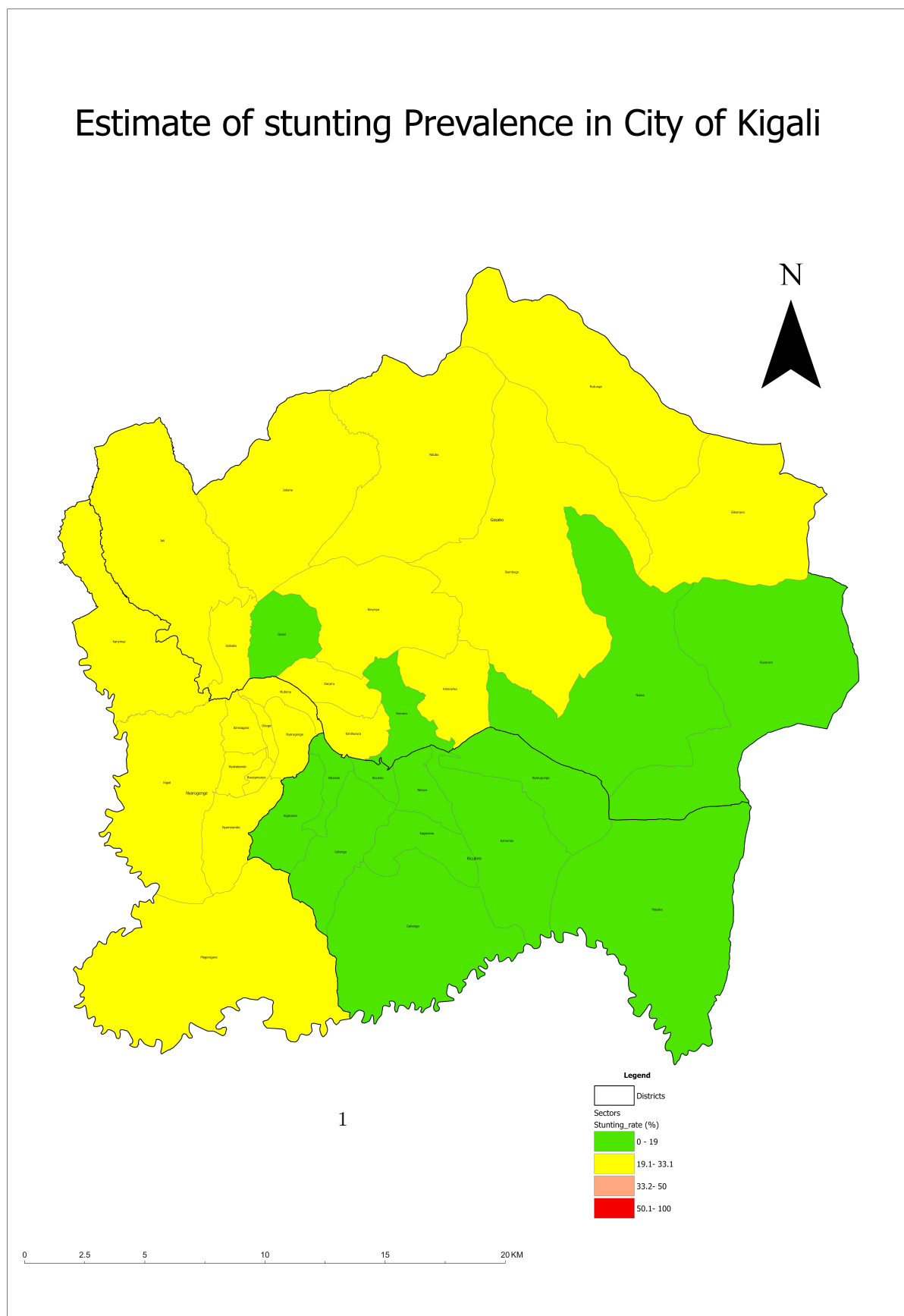

Supplement: Supplementary file 1 — Additional file 1. This manusrcipt has separate accompying supplementary files. [file 40795_2023_806_MOESM1_ESM.zip › Supplement_Figure_A1.pdf]

**Fig.A 2** Estimate of Stunting Prevalence in Eastern Province

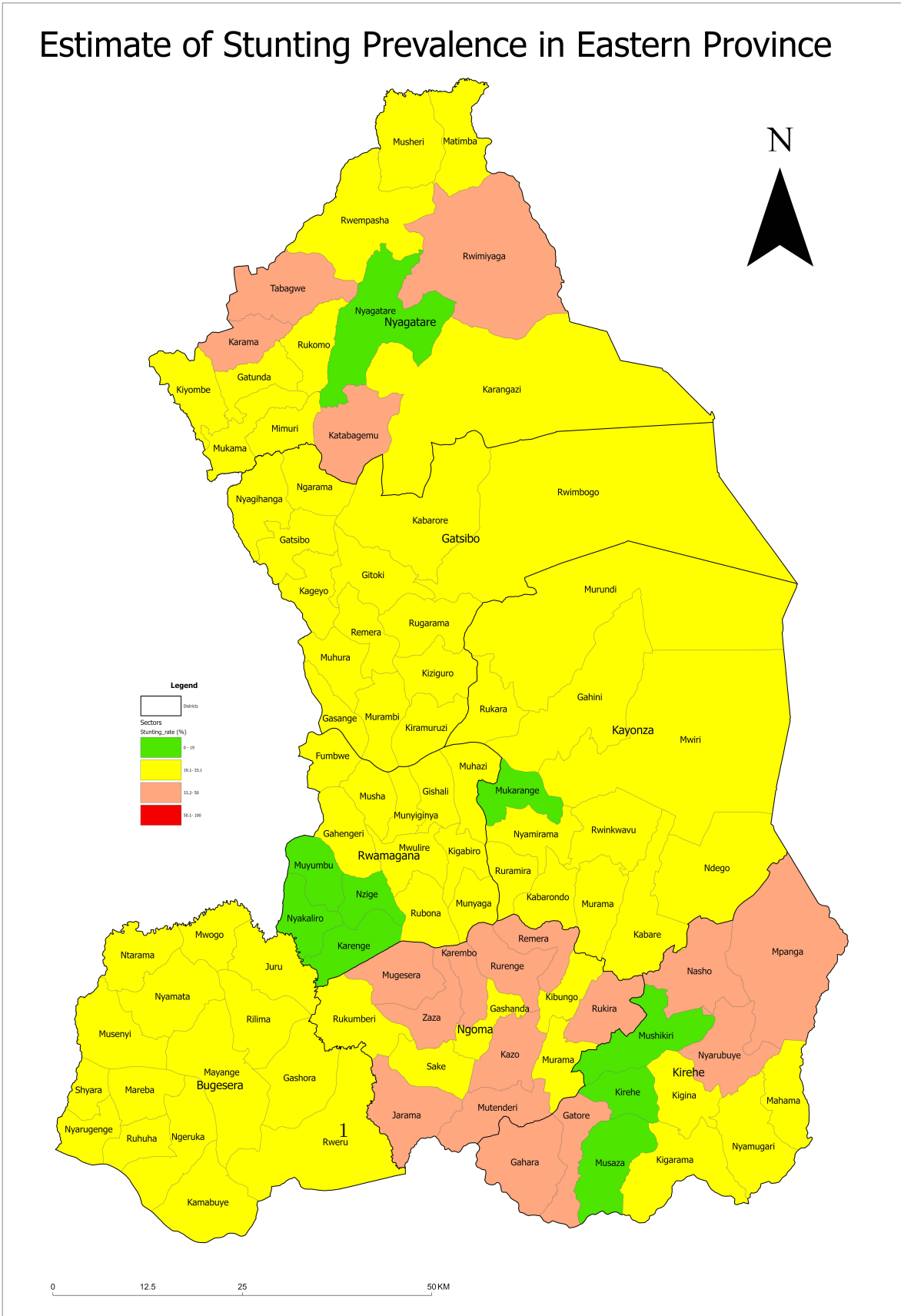

Supplement: Supplementary file 1 — Additional file 1. This manusrcipt has separate accompying supplementary files. [file 40795_2023_806_MOESM1_ESM.zip › Supplement_Figure_A2.pdf]

**Fig.A 3** Estimate of Stunting Prevalence in Southern Province

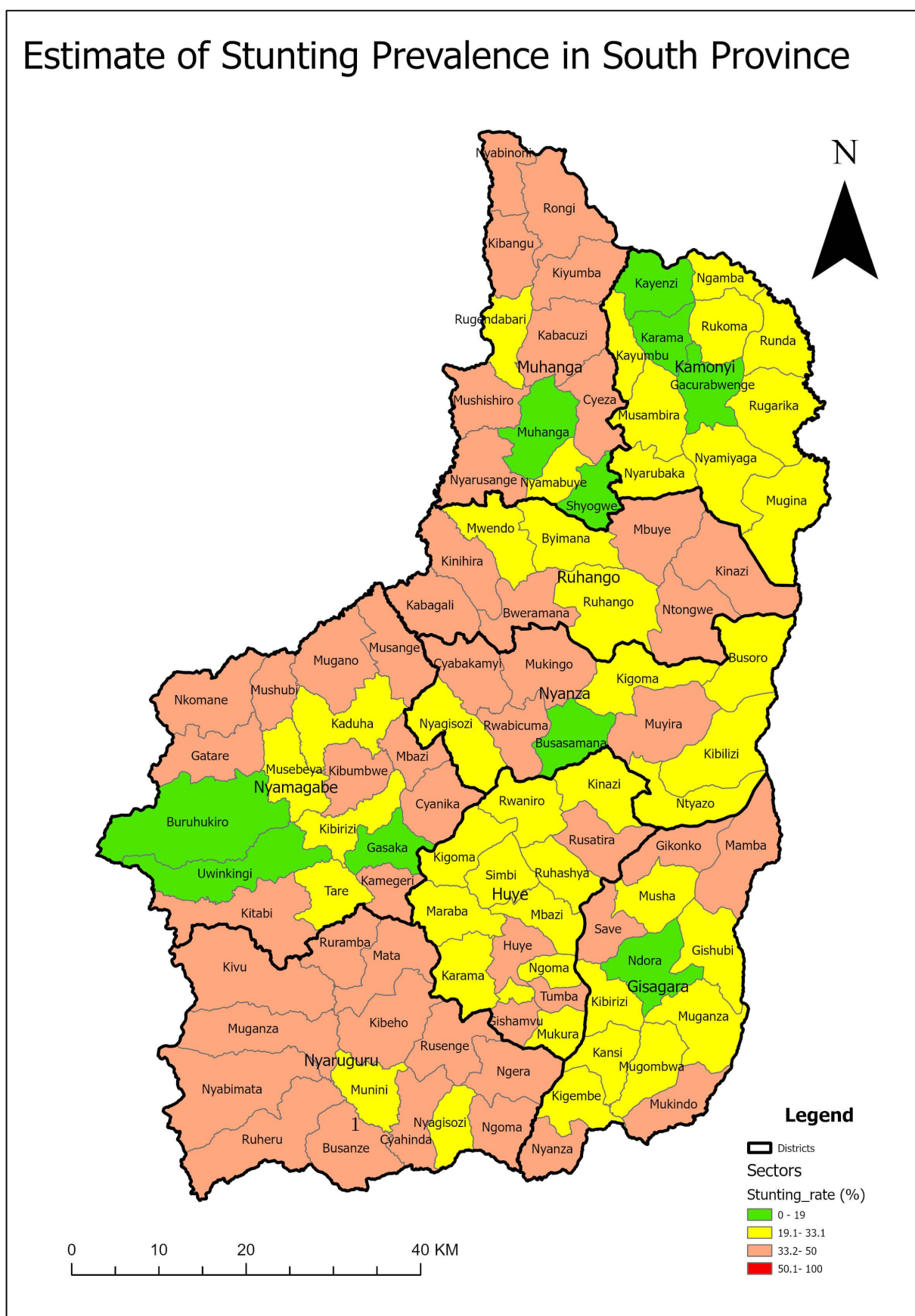

Supplement: Supplementary file 1 — Additional file 1. This manusrcipt has separate accompying supplementary files. [file 40795_2023_806_MOESM1_ESM.zip › Supplement_Figure_A3.pdf]

**Fig.A 4** Estimate of Stunting Prevalence in Northern Province

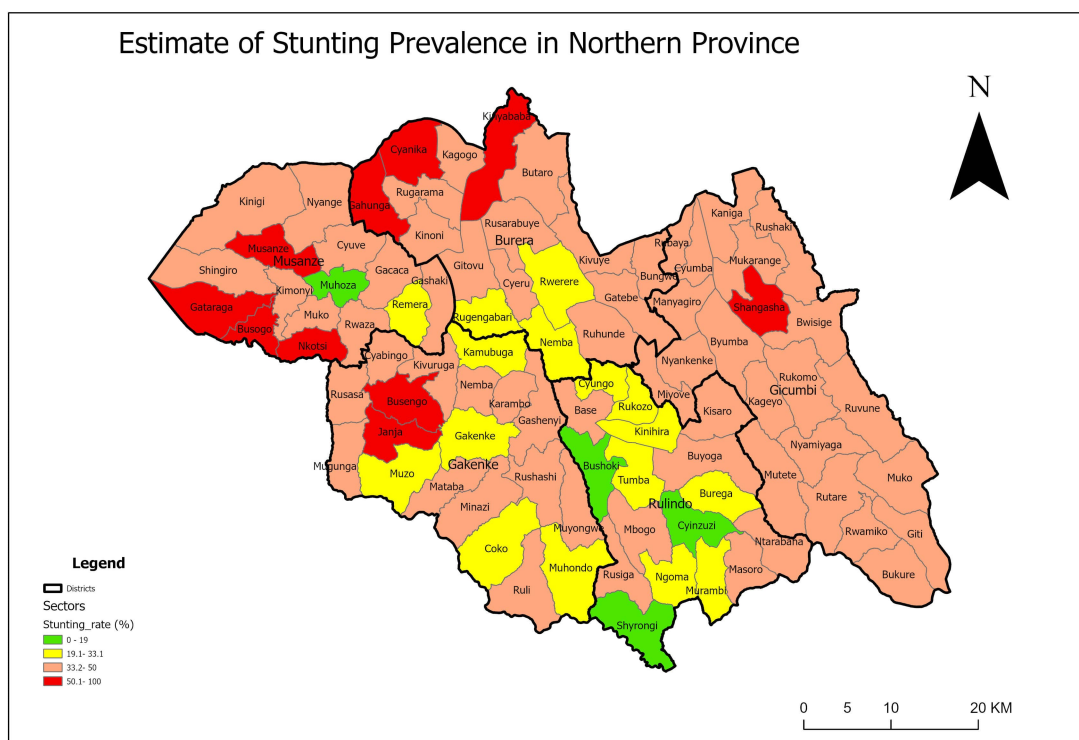

Supplement: Supplementary file 1 — Additional file 1. This manusrcipt has separate accompying supplementary files. [file 40795_2023_806_MOESM1_ESM.zip › Supplement_Figure_A4.pdf]

**Fig.A 5** Estimate of Stunting Prevalence in Western Province

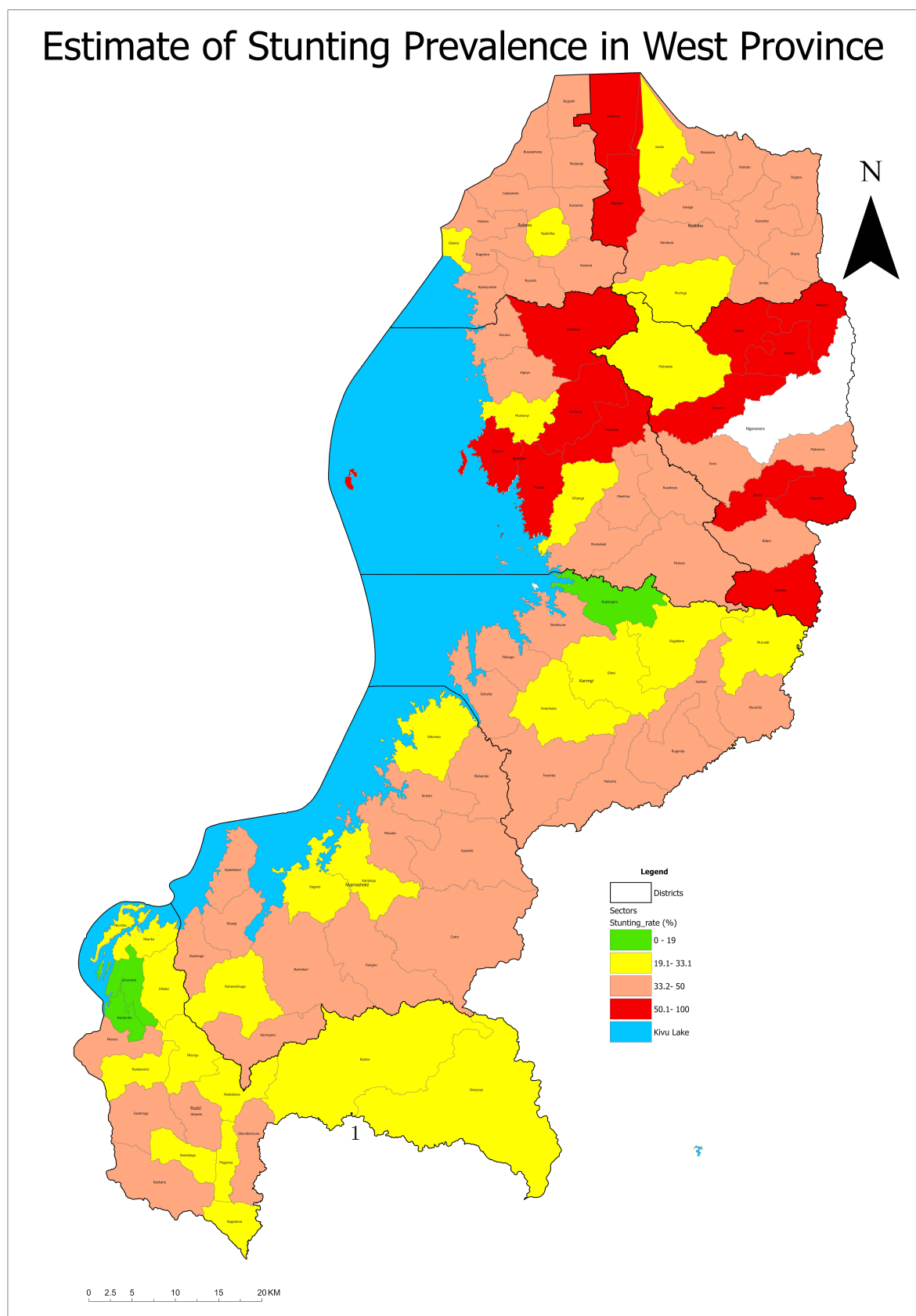

Supplement: Supplementary file 1 — Additional file 1. This manusrcipt has separate accompying supplementary files. [file 40795_2023_806_MOESM1_ESM.zip › Supplement_Figure_A5.pdf]
